# Limonin alleviates pyroptosis and inflammatory responses in cardiomyocytes of myocardial ischemia-reperfusion mice by inhibiting the caspase-3/GSDME pathway

**DOI:** 10.3389/fimmu.2026.1704424

**Published:** 2026-04-29

**Authors:** Jianlong Li, Zhe Li, Pengran Wang, Zhian Jiang

**Affiliations:** 1 Department of Cardiology, Hebei Medical University Third Hospital, Shijiazhuang, China; 2 Department of Cardiology, Affiliated Hospital of Hebei University, Baoding, China; 3 Department of Nephrology, Affiliated Hospital of Hebei University, Baoding, China

**Keywords:** caspase-3/GSDME signaling pathway, inflammation, limonin, myocardial ischemia/reperfusion injury, pyroptosis

## Abstract

**Purpose:**

Myocardial ischemia-reperfusion (I/R) is characterized by myocardial cell death and exacerbated inflammatory responses that seriously affect cardiac function and patient prognosis. Limonin is a natural compound extracted from citrus fruits and has various biological activities, including anti-inflammatory and antioxidant activities. However, the role and mechanism of action of limonin in myocardial I/R injury remain unclear. This study aimed to explore the effects and underlying mechanisms of action of limonin on myocardial cell pyroptosis and inflammatory responses in mouse myocardial I/R injury.

**Methods:**

By analyzing the GSE225105 dataset in the Gene Expression Omnibus database, differentially expressed genes in I/R injury were screened and combined with the target genes of limonin in the Comparative Toxicogenomics Database. In the *in vitro* experiments, The oxygen-glucose deprivation/reoxygenation (OGD/R) model was used to simulate I/R injury in *in vitro* experiments.

**Results:**

Limonin significantly inhibited OGD/R-induced pyroptosis and the release of inflammatory factors in AC16 myocardial cells, and these effects could be reversed by the caspase-3/gasdermin E (GSDME) pathway activator triclabendazole. Using *in vivo* experiments, we established a myocardial I/R model in C57BL/6 mice and found that limonin pretreatment improved cardiac function-related indicators, reduced myocardial tissue damage and inflammatory responses, inhibited apoptosis, and reduced myocardial fibrosis. This mechanism is closely related to the inhibition of caspase-3/GSDME pathway activation.

**Conclusion:**

This study revealed the protective effects of limonin in reducing myocardial I/R injury by inhibiting the caspase-3/GSDME pathway, thereby providing a theoretical basis for the development of limonin-based myocardial protection strategies.

## 1 Introduction

Cardiovascular diseases pose a serious threat to human health. Among them, acute myocardial infarction and the subsequent myocardial ischemia-reperfusion (I/R) injury are critical pathological processes leading to the deterioration of cardiac function (1). In I/R injury, programmed cell death interacts with inflammatory responses to collectively determine the fate of cardiomyocytes. Pyroptosis, as a pro-inflammatory form of programmed cell death, has been confirmed in recent years to participate in the progression of I/R injury in recent years (2). In addition to the classical inflammasome-caspase-1 pathway, recent studies have revealed that caspase-3 can trigger pyroptosis by cleaving gasdermin E (GSDME): activated caspase-3 cleaves full-length GSDME to generate the pore-forming N-terminal fragment (GSDME-N), which perforates the cell membrane, leading to cell swelling and rupture, and the release of inflammatory factors such as IL-18, thereby amplifying the local inflammatory response (3). This pathway was initially identified in chemotherapy-induced cell death and has subsequently been observed to play a pathological role in diseases such as nerve injury and atherosclerosis (4, 5). In recent years, studies have confirmed the involvement of the caspase-3/GSDME pathway in myocardial I/R injury (6, 7); however, its specific regulatory mechanisms and upstream signals under these pathological conditions have not yet been fully elucidated. Therefore, an in-depth investigation into the role of this pathway in myocardial I/R and its regulatory network will help to further clarify the molecular mechanisms of pyroptosis and provide a new theoretical basis and potential targets for intervening in myocardial injury.

Natural products have always been an important source for new drug development due to their structural diversity and rich biological activities (8). Limonin, a tetracyclic triterpenoid compound isolated from plants of the Citrus genus, is one of the main components responsible for the bitter taste ofin citrus fruits (9). Recent studies have shown that limonin possesses various pharmacological activities, including anti-inflammatory, antioxidant, and anti-apoptotic effects, demonstrating protective roles in multiple disease models (10). Notably, the latest research reports that limonin can mediate cardiac repair following myocardial infarction by regulating citrate metabolism, suggesting its potential value in the treatment of heart diseases (11). Furthermore, limonin has been confirmed to inhibit caspase-3 activation and the release of inflammatory factors in various cell models (12, 13), processes that are closely related to the caspase-3/GSDME pyroptosis pathway (14). Based on this background, we hypothesized that limonin might exert its protective effects against myocardial I/R injury by regulating the caspase-3/GSDME pathway to influence cardiomyocyte pyroptosis. However, to date, no studies have reported on the regulation of this pathway by limonin in the context of I/R injury. Therefore, this study aimed to investigate the protective effect of limonin against myocardial I/R injury and its regulatory mechanism involving the caspase-3/GSDME pathway. The findings of this study could provide a new therapeutic target and candidate drug for the prevention and treatment of myocardial I/R injury.

Programmed cell death is crucial for maintaining homeostasis and for responding to pathological stimuli. Pyroptosis is a more recently identified type of programmed cell death, has received considerable attention. The caspase family of proteins plays a core regulatory role in apoptosis, with caspase-3 being the key executor (15). Recent studies have demonstrated that it is also closely associated with pyroptosis. GSDME is inactive in normal cells, and its activation by caspase-3 can cleave it and trigger pyroptosis, leading to cell membrane perforation, cell rupture, the release of inflammatory factors, thus triggering an inflammatory response (5). Under physiological conditions, precise regulation of pyroptosis is beneficial to the body. However, during the occurrence and development of diseases, abnormal activation of the caspase-3/GSDME signaling pathway can lead to excessive pyroptosis, aggravating tissue damage and inflammation. For example, Bnip3 mediates doxorubicin-induced pyroptosis via caspase-3/GSDME (16). However, the involvement of the caspase-3/GSDME signaling pathway in I/R has not been reported to date.

## 2 Methods

### 2.1 Screening of differentially expressed I/R genes through online databases

The Gene Expression Omnibus (GEO) database (https://www.ncbi.nlm.nih.gov/geo/) was used to obtain the public expression data. The dataset GSE225105 was used to explore the differential expression between two groups: myocardial I/R and control (Ctrl) groups. The “limma” package was used to analyze the differential expression of the two groups (17). Using the R packages “FactoMineR” and “factoextra”, principal component analysis (PCA) was performed on the two groups of samples based on the differentially expressed genes (18). Based on the differential analysis of GSE225105, gene logFC ranking was obtained, and the R package “clusterProfiler” was used to perform gene set enrichment analysis (GSEA) for all genes (19).

### 2.2 Acquisition of limonin targets

The CTD database (https://ctdbase.org/) was used to screen for the action targets of limonin. Specifically, “Limonin” was used as the keyword to search the CTD database to retrieve target genes with documented interactions with this compound. The search was performed using the database’s default settings without additional filtering criteria. According to the default filtering logic of CTD, interactions labeled as “does not affect” were automatically excluded, retaining only those with clear functional effects, such as “increases,” “decreases,” or “affects.” All interaction types (e.g., binding, expression regulation) were included without restriction by type. The species were not limited, and all curated literature evidence was integrated, with any interaction supported by at least one reference being included. Results were sorted in descending order by reference count by default and displayed at 50 records per page. This strategy ensured the originality and reliability of the data while maximizing the retention of complete, literature-supported interaction information.

### 2.3 Kyoto encyclopedia of genes and genomes, gene ontology functional enrichment, and venn diagram approaches

The R package “clusterProfiler” was used to perform GO functional enrichment analysis and KEGG pathway enrichment analysis on the differentially expressed genes obtained from the GSE225105 database, aiming to investigate the involved biological functions and signaling pathways. The significance threshold for enrichment analysis was set at adjusted P-value (p.adjust) < 0.05, and the Benjamini−Hochberg method was applied for multiple testing correction. The analysis results were visualized using bubble plots to display the top significant enrichment terms, with significance indicated by p.adjust values (20).

### 2.4 Protein-protein interaction network analysis based on GeneMANIA

The GeneMANIA database (http://genemania.org/) was used to perform a protein-protein interaction network analysis on the two intersecting genes obtained. The species was restricted to Mus musculus, and the analysis was conducted using the database default settings (no minimum interaction score or network type filters were specified). Under this default mode, GeneMANIA automatically integrates all types of mouse association networks (including physical interactions, co-expression, co-localization, etc.) and employs a built-in machine learning weighting algorithm to identify and display the 20 genes most closely associated with the input genes, thereby constructing a functional association network.

### 2.5 Molecular docking

The X−ray diffraction protein three−dimensional structure of human Caspase−3 (Protein Data Bank [PDB] code: 1qx3) was downloaded from the PDB database. The protein structure was processed using AutoDock Tools: water molecules were removed, hydrogen atoms were added, charges were calculated, non−polar hydrogens were merged, and the three−dimensional structure was further optimized. The small−molecule ligand limonin was obtained in MOL2 format from the Traditional Chinese Medicine Systems Pharmacology Database and converted to PDB format using AutoDock Tools, followed by conversion to PDBQT format for docking. The docking grid box was centered at coordinates (center_x = 6.663, center_y = 36.66, center_z = 2.632) with dimensions of 64.05 Å × 64.05 Å × 64.05 Å (size_x, size_y, size_z). Molecular docking was then performed using AutoDock Vina (The Scripps Research Institute, La Jolla, CA, USA) (21). Visualization was carried out using PyMOL 2.1.0 (Schrödinger, Inc., New York, NY, USA) to generate the three−dimensional interaction diagram (22).

### 2.6 Cell culture

Human cardiomyocytes AC16 cells were purchased from Wuhan Procell Life Science & Technology Co. Ltd. (Wuhan, China). The cells were retrieved from liquid nitrogen, resuspended, and cultured in 1640 medium (a mixture of 10% fetal bovine serum [G8005; Wuhan Servicebio Technology Co. Ltd, Wuhan, China] and 1% penicillin-streptomycin [G4003 and G4532, Wuhan Servicebio Technology Co. Ltd.]). Limonin (HY-17411) and triclabendazole (CGA89317) were purchased from MedChem Express. The cells were divided into four groups: control, oxygen-glucose deprivation/reoxygenation (OGD/R), OGD/R + limonin, and OGD/R + limonin + triclabendazole. Among them, triclabendazole is an activator of the caspase-3/GSDME signaling pathway (23). The OGD/R model was established by placing cultured cardiomyocytes under sugar-free, serum-free, and hypoxic conditions (1% O_2_, 5% CO_2_, and 94% N_2_) in an incubator for 6 h to simulate the ischemic state. After oxygen-glucose deprivation, the medium was replaced with normal complete medium and the cells were placed in a normoxic incubator to simulate reoxygenation. Limonin (50 μmol/L) and triclabendazole (40 μmol/L) were added to AC16 cells and incubated for 24 h before OGD/R modeling. Triclabendazole activates the caspase-3/GSDME pathway (24).

### 2.7 Cell Counting Kit-8 assay

Cardiomyocytes were prepared in a good growth state by adding 2 mL of trypsin (G4001; Wuhan Servicebio Technology Co. Ltd.) and incubating the cells for 2 min. Next, 2 mL of 1640 medium was added to resuspend the cells in centrifuge tubes at 1200 × *g* for 3 min. RPMI 1640 medium (10 mL) was added to resuspend the cells, and the cell density was calculated using a cell counter. The cells were seeded in 96-well plates at a density of 5000 AC16 cells per well. Then, 10 μL of CCK8 solution (G1613; Wuhan Servicebio Technology Co. Ltd.) was added to each well, and the cells were incubated for 4 h. The absorbance (OD value) of each well was measured at 450 nm using a microplate reader.

### 2.8 Hoechst 33258 experiment

AC16 cells were grown in the exponential growth phase. According to the experimental design, cells were treated with trypsin, and the cell suspension was collected. Cells (1 × 10^5^ cells/well) were seeded in 6-well plates and incubated overnight. The cells were then fixed with 4% paraformaldehyde (G1101; Wuhan Servicebio Technology Co. Ltd.) for 30 min the next day. They were then rinsed with phosphate-buffered saline (PBS) three times for 5 min each time. Hoechst 33258 staining solution (G1011; Wuhan Servicebio Technology Co. Ltd.) was added to cover the cells, which were then incubated at 25°C in the dark for 15 min. The Hoechst 33258 staining solution was aspirated, and PI staining solution (G1021; Wuhan Servicebio Technology Co. Ltd.) was added; cells were then incubated at 25°C in the dark for 10 min. The cells were observed under a fluorescence microscope (Olympus Corporation, Tokyo, Japan) and photographed. The images were retained for subsequent statistical analyses.

### 2.9 Flow cytometry

The cells were collected and cultured cardiomyocytes were treated as described above. The cells were digested with trypsin, and the cell suspension was collected. Annexin V-FITC and PI staining solution (5 μL of each) were added to the cells according to the instructions of the apoptosis detection kit (G1511; Wuhan Servicebio Technology Co. Ltd.). The mixture was then incubated at 25°C in the dark for 20 min. After incubation, the cells were resuspended in an appropriate amount of binding buffer. The samples were then subjected to flow cytometry.

### 2.10 Enzyme-linked immunosorbent assay (ELISA) experiments

AC16 cells were treated as described previously, and the supernatant of the AC16 cell culture was collected. Blood was also collected, placed in anticoagulation tubes, and centrifuged at 5000 × *g* for 30 min to obtain the serum. Mouse myocardial tissue was also collected, and an appropriate amount of tissue homogenate was added according to the requirements of the kit and centrifuged after processing to obtain the supernatant. The diluted coating antibody (100 μL/well) was added to each well of a 96-well microplate and incubated overnight at 4 °C. The coating solution was discarded, and the wells were washed three times with the washing solution. Blocking solution (200 μL) was added to each well and cells were then incubated at 25°C for 2 h. The blocking solution was discarded and the wells were washed three times. The standards and samples (100 μL/well) were added to the respective wells, and the plate were sealed with a sealing film and incubated at 25°C for 2 h. The liquid in each well was discarded, and the wells were washed five times. Diluted detection antibody (100 μL) was then added to each well, and the cells were incubated at 25°C for 1 h. Diluted enzyme-labeled antibody (100 μL) was added to each well, and the cells were incubated at 25°C for 30 min. Chromogenic agent (100 μL) was added to each well, and the cells were further incubated at 25°C in the dark for 30 min. Fifty μL of a termination solution were added to each well to terminate the chromogenic reaction. The OD of each well was measured at 450 nm using a microplate reader. For the manufacturers and product numbers of the ELISA kits, please refer to **Supplementary Table 1**.

### 2.11 Animal experiments

Forty male C57BL/6 mice aged 6–8 weeks and weighing 20–28 g were purchased from Vital River Laboratory Animal Technology Co, Ltd. (Beijing, China) and housed at the Experimental Animal Center of the Medical Department of Hebei University. The mice were randomly assigned to 4 groups (n = 10 per group) using a random number table. The I/R + limonin and I/R + limonin + triclabendazole groups were intraperitoneally injected with 40 mg/kg limonin once daily for seven days before surgery (25). The I/R + limonin + triclabendazole group was intraperitoneally injected with 100 mg/kg triclabendazole one day before surgery. The sham and I/R groups were injected with equal volumes of normal saline (24).

For the I/R model, mice were anesthetized by intraperitoneal injection of 70 mg/kg pentobarbital sodium (WKQ-0028128; Sichuan Vicky Biotechnology Co. Ltd, Shanghai, China). After anesthesia, an electrocardiogram (Shanghai Yuyan Biotechnology Co. Ltd, Shanghai, China) was connected to collect the standard lead II. After tracheal intubation, a small animal ventilator was connected for mechanical ventilation, with a tidal volume of 0.6 to 0.8 mL/min and frequency of 100 to 120 times/min; I:E = 2:1. Thoracotomy was performed between the 3rd and 4th intercostal spaces on the left chest. Heparin sodium (500 IU/kg) was intraperitoneally injected to maintain reperfusion after occlusion. The pericardium was cut open to expose the left atrial appendage and pulmonary artery cone. A 5.0 prolene suture was threaded at the starting part of the anterior descending branch of the coronary artery for standby. In the other groups, except for the sham operation group, a rubber band was placed at the bottom, and the anterior descending branch of the coronary artery was ligated with double lines. Successful occlusion leading to myocardial ischemia was marked by a significantly elevated ST segment and tall T wave (> 0.12 mV) on electrocardiography, darkened myocardial color below the ligation line, and decreased myocardial contractility. After 30 min of ischemia, the ligation line was loosened to restore blood flow. Reperfusion was allowed to proceed for 2 h, after which cardiac ultrasound detection was performed (26). Following ultrasound examination, all mice were maintained under observation for an additional 22 h (totaling 24 h of reperfusion) before being euthanized. Mice were euthanized by cervical dislocation after ocular ischemia under anesthesia (intraperitoneal injection of 70 mg/kg pentobarbital), and myocardial tissues were collected for subsequent experiments and pathological detection.

### 2.12 Cardiac ultrasonography

After the mice underwent I/R surgery, cardiac ultrasound detection was performed using a gaseous anesthetic machine (RWD Life Science Co. Ltd, Shenzhen, China) with 1.5–2.5% isoflurane (A425890; Sangon Biotech Co. Ltd, Shanghai, China). Hair was removed from the chest using a depilatory cream, and the animals were placed on a heating platform connected to an ultrasound system to measure their electrocardiogram and respiratory rate. The echocardiography system (Xuzhou Beiersi Electronic Technology Co. Ltd, Xuzhou, China) with a 30-MHz imaging sensor was used for detection, and parameters including heart rate (HR), ejection fraction (EF, %), fractional shortening (FS, %), left ventricular end-diastolic diameter (LVEDD), left ventricular end-systolic diameter (LVESD), left ventricular ejection fraction (LVEF), and left ventricular fractional shortening (LVFS) were measured (27). Images and data were retained for subsequent experimental analyses.

### 2.13 Hematoxylin and eosin staining

Mouse myocardial tissues were sampled and quickly fixed in 4% paraformaldehyde for 48 h. Gradient dehydration was performed successively in 70%, 80%, 90%, 95%, and 100% ethanol (E111964; Shanghai Aladdin Biochemical Technology Co. Ltd, Shanghai, China) for 2 h at each level. The tissues were rendered transparent in xylene (X112050; Shanghai Aladdin Biochemical Technology Co. Ltd.), twice for 30 min each time. The tissue samples were then placed in melted paraffin and allowed to soak at 60 °C in an oven for 3 h. The paraffin-soaked tissue was placed in an embedding box, paraffin was injected, and then the tissue was cooled and solidified. The tissue was then sliced into 5 μm thin sections with a microtome and mounted on glass slides. The slides were baked in an oven at 60 °C for 3 h. Sections were successively passed through a xylene and ethanol gradient in water. The sections were stained with hematoxylin staining solution (H104302; Shanghai Aladdin Biochemical Technology Co. Ltd.) for 10 min. The cells were then rinsed with tap water until the nuclei turned blue. Sections were then placed in an eosin staining solution (E489856; Shanghai Aladdin Biochemical Technology Co. Ltd.) for 1 min. The samples were successively passed through a gradient of ethanol and xylene to ensure dehydration and transparency. The sections were then mounted with neutral gum and observed under a microscope.

### 2.14 Masson staining

Sections were stained with Weigert’s hematoxylin solution for 10 min. After washing with water, the cells were differentiated using 1% hydrochloric acid and alcohol. The samples were washed with water until the color turned blue and then were washed with distilled water. The sections were then stained with Ponceau acid fuchsin solution for 10 min and soaked briefly in a 2% aqueous glacial acetic acid solution. The cells were differentiated using a 1% phosphomolybdic acid aqueous solution for 5 min. The samples were stained with aniline blue solution for 5 min without washing with water, then soaked briefly in a 0.2% aqueous glacial acetic acid solution. The samples were dehydrated with 95% alcohol and absolute alcohol, made transparent with xylene, and sealed with neutral gum.

### 2.15 Terminal deoxynucleotidyl transferase dUTP nick end labeling staining

Apoptotic cells in myocardial tissue sections were detected using a TUNEL assay kit (E-CK-A331; Wuhan Elabscience Biotechnology Co. Ltd, Wuhan, China) according to the manufacturer’s instructions. Briefly, after dewaxing and rehydration, the sections were incubated with proteinase K (20 μg/mL) at 37 °C for 30 min. Following three washes with PBS for 5 min each, the sections were incubated with the TUNEL reaction mixture (containing terminal deoxynucleotidyl transferase and labeled dUTP) in a humidified chamber at 37 °C for 60 min. The sections were then washed three times with PBS and incubated with Converter-POD (horseradish peroxidase-labeled streptavidin) in a humidified chamber at 37 °C for 30 min. After another three washes with PBS, the sections were stained with 3,3’-diaminobenzidine (DAB) chromogenic solution for 2–5 min, and the reaction was monitored under a microscope to avoid overdevelopment. Finally, the sections were counterstained with hematoxylin, dehydrated, cleared, and mounted. Five random fields per section were observed under a light microscope (Olympus Corporation, Tokyo, Japan), and the percentage of TUNEL-positive cells was calculated.

### 2.16 RT-qPCR assay

Myocardial cells or tissue samples were collected and processed according to the instructions of the RNA extraction kit (19211ES60; Yeasen Biotechnology Co. Ltd, Shanghai, China) to extract total RNA and determine its concentration and purity. For cDNA synthesis, an appropriate amount of RNA was extracted using a reverse transcription kit (11155ES; Yeasen Biotechnology Co. Ltd.) to reverse transcribe RNA into cDNA. The reaction conditions were set according to the kit instructions: incubation at 42 °C for 30 min and heating at 85 °C for 5 min to inactivate the reverse transcriptase. Specific primers for the target and reference genes were designed for qPCR. The qPCR reaction system was prepared as required by the kit (11175ES; Yeasen Biotechnology Co. Ltd.) and added to 96-well plates. The reaction proceeded under the following conditions: pre-denaturation (95 °C, 4 min), denaturation (95 °C, 13 s), annealing (set according to the annealing temperature of the primers, 16 s), and extension (72 °C, 16 s) performed for 40 cycles. After the reaction was complete, the cycle threshold (Ct) value was obtained using the software provided with the instrument. The relative expression level of the target gene was calculated using the 2^(-ΔΔCt) method, where ΔCt = Ct value of the target gene - Ct value of the reference gene, and ΔΔCt = ΔCt of the experimental group - ΔCt of the control group. The RT-qPCR primer sequences are listed in **Supplementary Table 2**.

### 2.17 Western blotting

Myocardial cells or tissues were extracted, and appropriate amounts of tissue and cell lysis buffer were added. Samples were incubated on ice for 30 min. Protein concentration was determined using a BCA protein quantification kit (20201ES; Yeasen Biotechnology Co. Ltd.). Protein loading buffer (20315ES; Yeasen Biotechnology Co. Ltd.) was added and samples were incubated at 97 °C in a water bath (Changzhou Yineng Experimental Instrument Factory, Changzhou, China) for 30 min. The treated samples were added to the gel-loading wells, and electrophoresis was performed at 120 V for 90 min. After electrophoresis, the membranes were subjected to a constant current of 260 mA for 40 min. After transfer, the membrane was placed in a blocking solution (5% skim milk powder) and blocked at 25°C for 2 h. The membranes were then placed in diluted primary antibody solution and incubated at 4 °C overnight. The membrane was then washed three times with TBST for 15 min each time, placed in a diluted secondary antibody solution, and incubated at 25°C for 2 h. The membrane was then placed in a chemical chromogenic solution (36222ES; Yeasen Biotechnology Co. Ltd.) and incubated in the dark for 10 s. Images were obtained using a chemiluminescence imaging system (80551ES; Yeasen Biotechnology Co. Ltd.), and grayscale analysis of the bands was performed using ImageJ version 1.52a (National Institute of Health, Bethesda, MD, USA). The antibody information is presented in **Supplementary Table 3**.

### 2.18 Statistical analysis

All data were analyzed using SPSS version 26.0 (IBM Corp, Armonk, NY, USA). Continuous variables were expressed as mean ± standard deviation (SD) from at least five independent experiments (n = 5 for *in vitro* studies; n = 10 for *in vivo* studies). Normality of data distribution was assessed using the Shapiro–Wilk test, and homogeneity of variances was evaluated by Levene’s test. For comparisons between two groups, an unpaired two-tailed Student’s t-test was applied. For multiple group comparisons, one−way analysis of variance (ANOVA) was performed followed by Tukey’s honestly significant difference (HSD) *post hoc* test when variances were homogeneous; if homogeneity of variances was violated, the Welch ANOVA with Games–Howell *post hoc* test was used. In experiments where only comparisons with the control group were of interest, Dunnett’s *post hoc* test was employed. A value of *P* < 0.05 was considered statistically significant. All statistical tests and corresponding *P*-values are indicated in the figure legends.

## 3 Results

### 3.1 Screening of candidate I/R genes and limonin targets

**Figure 1:**
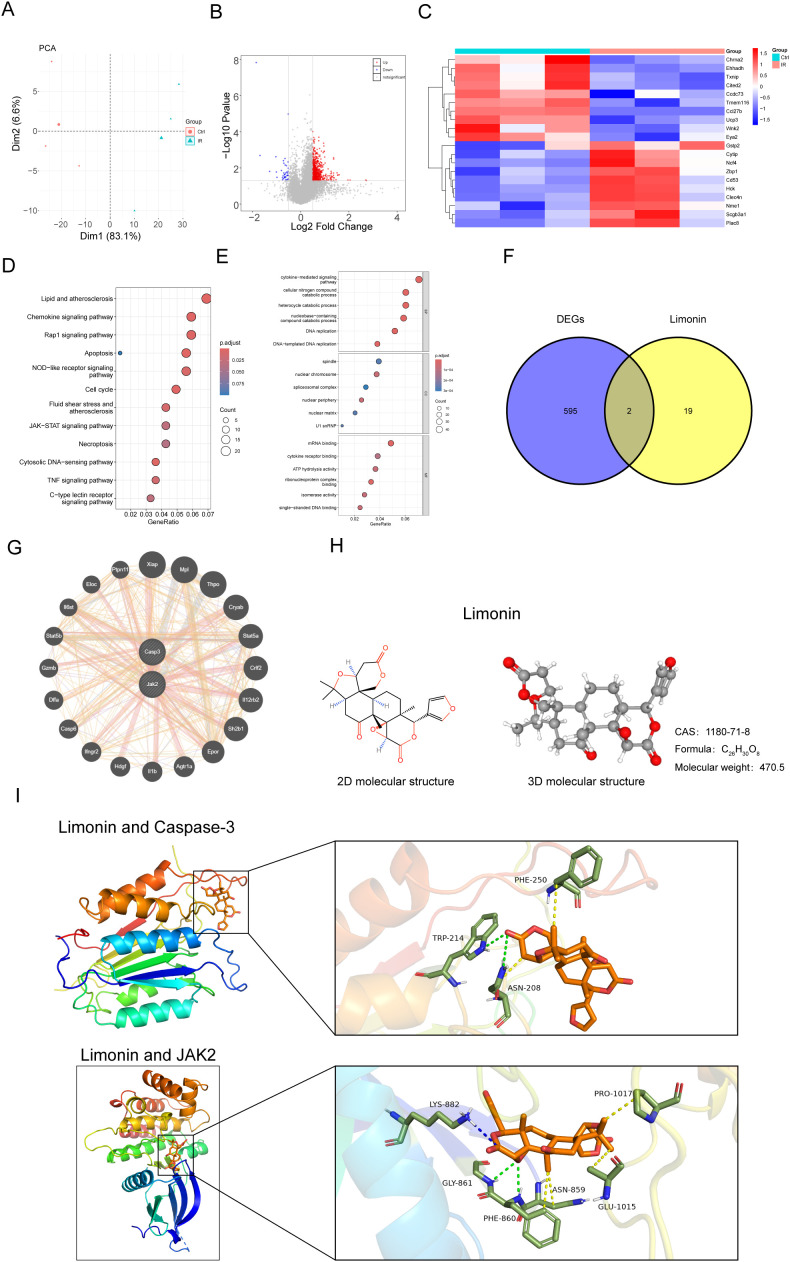
Differential gene analysis of GSE225105 and potential target analysis of limonin. **(A)** Principal component analysis (PCA). **(B)** Volcano plot of differentially expressed genes. **(C)** Heatmap of differentially expressed gene clustering. **(D)** KEGG functional enrichment analysis. **(E)** GO functional enrichment analysis. **(F)** Venn diagram of differentially expressed genes and drug targets. **(G)** Protein-protein interaction (PPI) analysis. **(H)** 2D and 3D chemical structures and relative molecular masses of limonin. **(I)** Molecular docking of limonin with caspase-3.

PCA was conducted using the data obtained from the GSE225105 dataset, and screening was performed according to |log FC| > 0.5 & *P*-value < 0.05. In total, 597 differentially expressed genes were identified, with 559 upregulated and 38 downregulated genes (**Figure 1A**). A volcano plot of the differentially expressed genes is shown in **Figure 1B**. The heatmap of differential gene clustering revealed that the top three genes with the most obvious upregulation in I/R were Plac8, scgb3a1, and Nme1, whereas the top three genes with the most obvious downregulation were Chma2, Ehhadh, and Txnip (**Figure 1C**). Subsequently, KEGG functional enrichment analysis revealed that the differentially expressed genes were mainly enriched in the lipid, atherosclerosis, chemokine signaling, and Rap1 signaling pathways (**Figure 1D**). GO functional enrichment analysis indicated the cytokine-mediated signaling pathway, cellular nitrogen compound catabolic process, and heterocycle catabolic process as the most enriched functions (**Figure 1F**). To further explore the potential therapeutic targets of limonin, we screened the intersection of limonin drug targets with the above differentially expressed genes using the venn diagram approach and identified two intersecting genes: those encoding JAK2 and caspase-3 (**Figure 1G**). To validate the interaction between limonin and these potential targets, molecular docking was performed. The docking results showed that limonin exhibited strong binding to caspase-3, with a binding energy of -8.6 kcal/mol. Limonin formed hydrogen bonds with the amino acid residues ASN208 and TRP214 of caspase-3, as well as hydrophobic interactions with PHE250 and ASN208 (**Figure 1I**). Given that the role of the JAK2 signaling pathway in I/R injury remains controversial based on literature reports and caspase-3 is a key executor of pyroptosis, we focused on caspase-3 in subsequent experiments.

### 3.2 Limonin counteracts the effect of the OGD/R model on the proliferation and pyroptosis of AC16 cells

**Figure 2:**
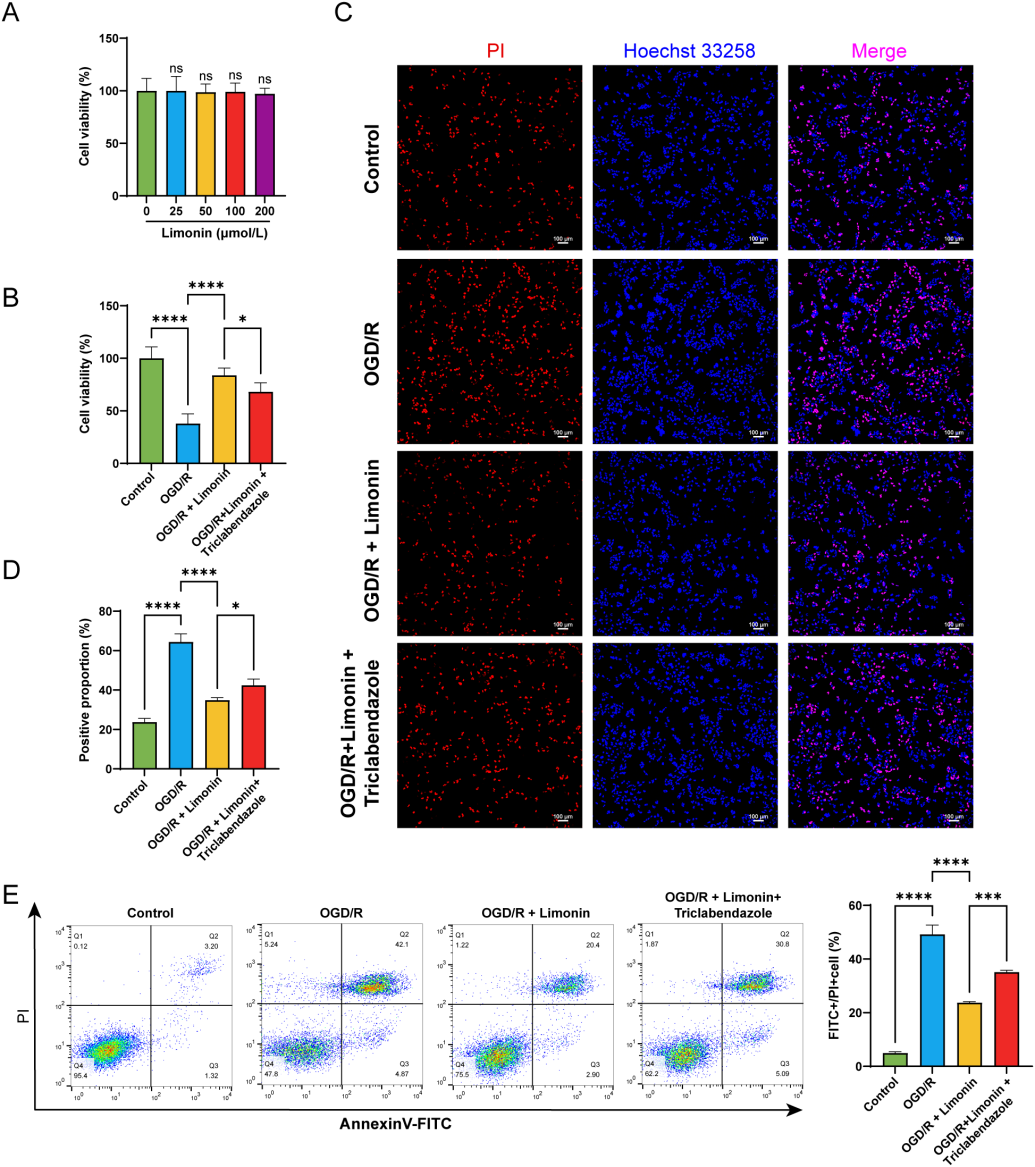
Limonin inhibits pyroptosis in cardiomyocyte AC16 cells. **(A)** CCK8 was used to detect the effects of limonin on the viability of AC16 cells after incubation with different concentrations of limonin for 24 h, and the OGD/R cell model was established after incubation (n = 5). **(B)** CCK8 was used to detect the effects of limonin (50 μmol/L) and triclabendazole (40 μmol/L) treatment for 24 h followed by OGD/R modeling on AC16 cells (n = 5). **(C)** PI/Hoechst 33258 double staining was used to detect the changes in pyroptosis (×100, Scale bar: 50 μm, n = 5). **(D)** Bar chart for statistical analysis of PI/Hoechst 33258. **(E)** Flow cytometry was used to detect the changes in pyroptosis (n = 5). ns represents no statistical significance compared with the control group, and * represents comparison between two groups (*P < 0.05, **P < 0.01, ***P < 0.001*, ****P < 0.0001).

To identify the non-toxic concentration of limonin in AC16 cells, we detected the effect of different concentrations of limonin on AC16 cells for 24 h using the CCK8 assay. We found that limonin had no effect on the viability of AC16 cells in the drug concentration range of 25–200 μmol/L (**Figure 2A**). Considering the results of *in vitro* experiments by other researchers, we selected a dose of 50 μmol/L of limonin for subsequent *in vitro* studies (28). We explored whether limonin could alleviate the damage caused by OGD/R in AC16 cells and whether its mechanism was achieved through the caspase-3/GSDME pathway by constructing an OGD/R model *in vitro* and combining treatment with triclabendazole (an activator of the caspase-3/GSDME pathway) and limonin. CCK8 assay results showed that OGD/R significantly reduced the viability of AC16 cells, while limonin pretreatment markedly attenuated this OGD/R-induced reduction in cell viability (**Figure 2B**).

To evaluate the effect of limonin on pyroptosis, Hoechst 33258/PI double staining and flow cytometry were performed. The addition of limonin significantly reduced the percentage of PI-positive cells in Hoechst 33258/PI staining (**Figure 2C**) and decreased the FITC/PI-positive cell population detected by flow cytometry (**Figure 2D**). However, these effects were reversed after the addition of triclabendazole. These results suggest that limonin inhibited OGD/R-induced pyroptosis in AC16 cells (**Figure 2E**).

### 3.3 Limonin improved cardiac function-related indicators in I/R model mice

**Figure 3:**
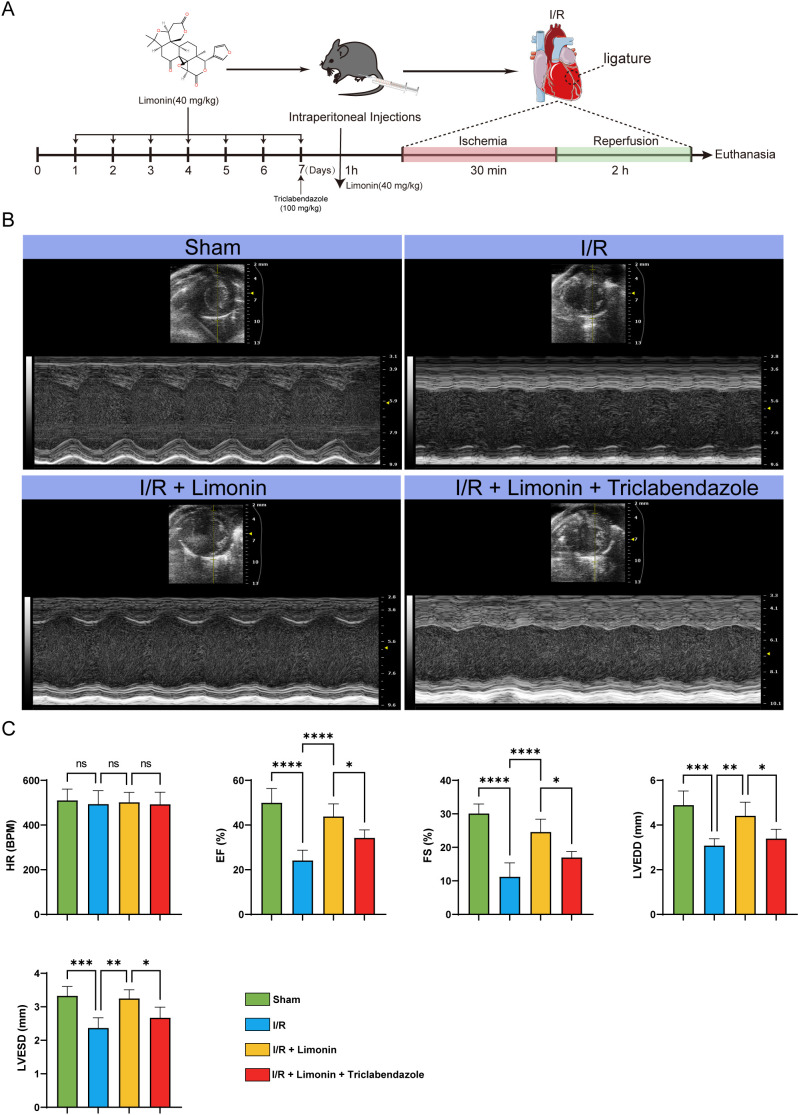
Limonin can improve cardiac function-related indicators in I/R mice. **(A)** Flowchart of I/R animal modeling and pretreatment with limonin and triclabendazole. **(B)** Small animal ultrasound detection (n = 10). **(C)** Detection of cardiac function-related indicators, including HR, EF (%), FS (%), LVEDD (mm), and LVESD (mm) (n = 10). ns represents no statistical significance between the two groups, and * represents comparison between the groups (*P < 0.05, **P < 0.01, ***P < 0.001*, ****P < 0.0001).

The method for establishing the I/R model and the pretreatment processes with limonin and triclabendazole are detailed in **Figure 3A**. Detection of cardiac function-related indicators showed that the heart rate of mice in the limonin-treated group was more stable and close to normal. In terms of cardiac systolic function indicators, EF (%) and FS (%) significantly increased in the limonin-treated group, indicating enhanced ejection capacity and shortening function of the heart. Regarding ventricular structural indicators, limonin treatment significantly reduced LVEDD (mm) and LVESD (mm), indicating that the inner diameters of the ventricles at the end of diastole and systole tended to be within the normal range (**Figures 3B, C**).

### 3.4 Limonin improved tissue damage and inflammatory responses in I/R model mice

**Figure 4:**
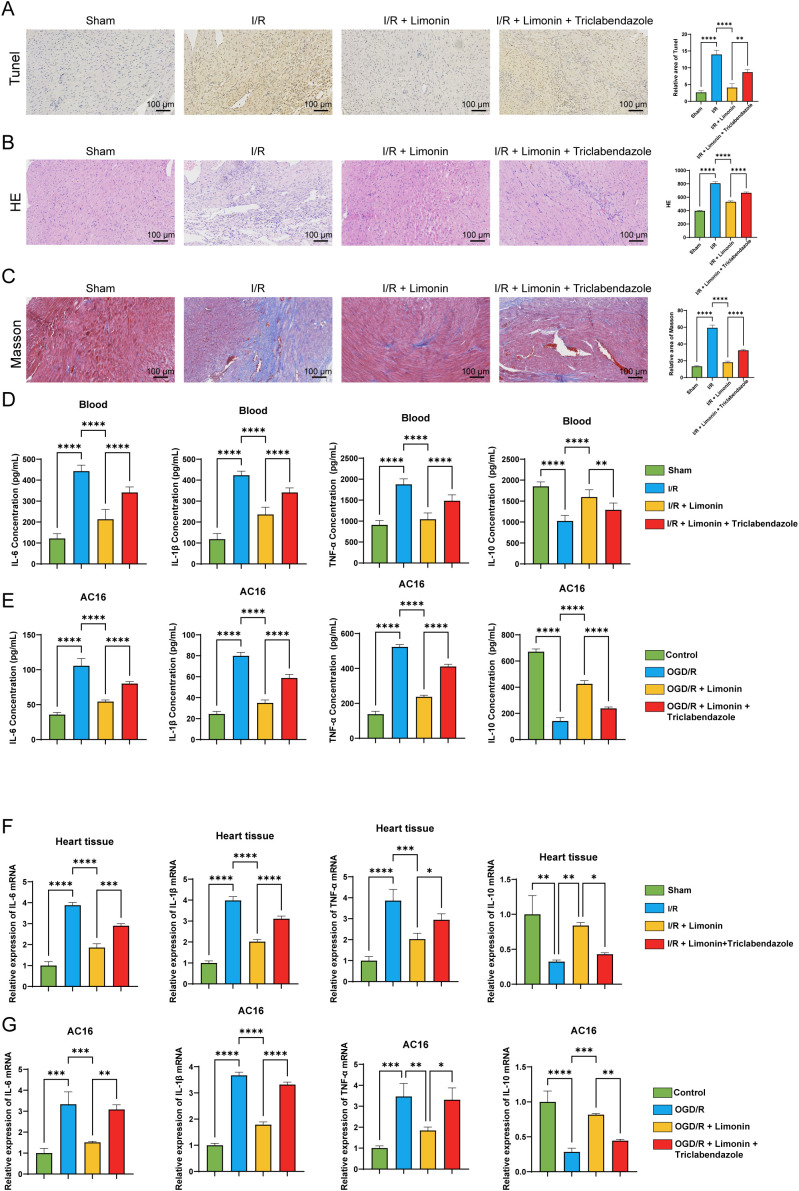
Limonin can improve tissue damage and inflammatory response in I/R mice. **(A)** TUNEL staining was used to detect the changes in myocardial tissue apoptosis (×100, Scale bar: 100 μm, n = 10). **(B)** HE staining was used to detect pathological changes in the myocardial tissue (×100, Scale bar: 100 μm, n = 10). **(C)** Masson staining was used to observe changes in the myocardial tissue (×100, Scale bar: 100 μm, n = 10). **(D)** ELISA was used to detect changes in inflammatory factors IL-6, IL-1β, TNF-α, and IL-10 in the supernatant of AC16 cells (n = 5). **(E)** ELISA was also used to detect changes in inflammatory factors IL-6, LI-1β, TNF-α, and IL-10 in mouse blood (n = 10). **(F)** Relative expression levels of IL-6, IL-1β, TNF-α, and IL-10 mRNA in mouse myocardial tissues detected by RT-qPCR. **(G)** Relative expression levels of IL-6, IL-1β, TNF-α, and IL-10 mRNA in AC16 cells detected by RT-qPCR. * represents comparison between two groups (*P < 0.05, **P < 0.01, ***P < 0.001*, ****P < 0.0001).

To further verify the pharmacological effects of limonin and triclabendazole, we observed cell apoptosis using TUNEL staining and found that cell apoptosis significantly increased in the I/R model, whereas limonin treatment significantly reduced the number of apoptotic cells. However, the number of apoptotic cells increased after the addition of triclabendazole, which reversed the antiapoptotic effects of limonin (**Figure 4A**). Although TUNEL staining is primarily used to detect apoptosis, DNA damage may occur during the late stages of pyroptosis. An increase in the number of TUNEL-positive cells may indicate pyroptosis (29). HE staining revealed severe histopathological damage and inflammatory cell infiltration in the I/R model mice. Limonin alleviated this damage and infiltration, whereas triclabendazole aggravated the damage and inflammation (**Figure 4B**). Masson’s trichrome staining indicated fibrosis in the I/R model. Limonin inhibited fibrosis, whereas triclabendazole reversed this inhibitory effect, leading to aggravation of fibrosis (**Figure 4C**).

ELISA was conducted to detect inflammatory indicators in the blood of the mice and in the supernatant of AC16 cells. It was found that the levels of pro-inflammatory factors interleukin (IL-6), IL-1ß, and tumor necrosis factor (TNF)-α increased in the I/R model while the level of the anti-inflammatory factor IL-10 decreased. Limonin reduced the levels of proinflammatory factors and increased those of IL-10, thereby exerting an anti-inflammatory effect. However, triclabendazole treatment increased the levels of the pro-inflammatory factor and decreased those of IL-10, reversing the anti-inflammatory effect of limonin (**Figures 4D, E**). RT-qPCR was performed to detect the mRNA expression levels of inflammatory factor mRNA in myocardial tissues and AC16 cells, and the results were consistent with those obtained using ELISA. mRNA expression of pro-inflammatory factors increased in the I/R model. Limonin and triclabendazole inhibited and increased their expression, respectively, further confirming the reversal of the pharmacological effects of limonin by triclabendazole (**Figures 4F, G**).

### 3.5 Limonin inhibited caspase-3/GSDME pathway activation

**Figure 5:**
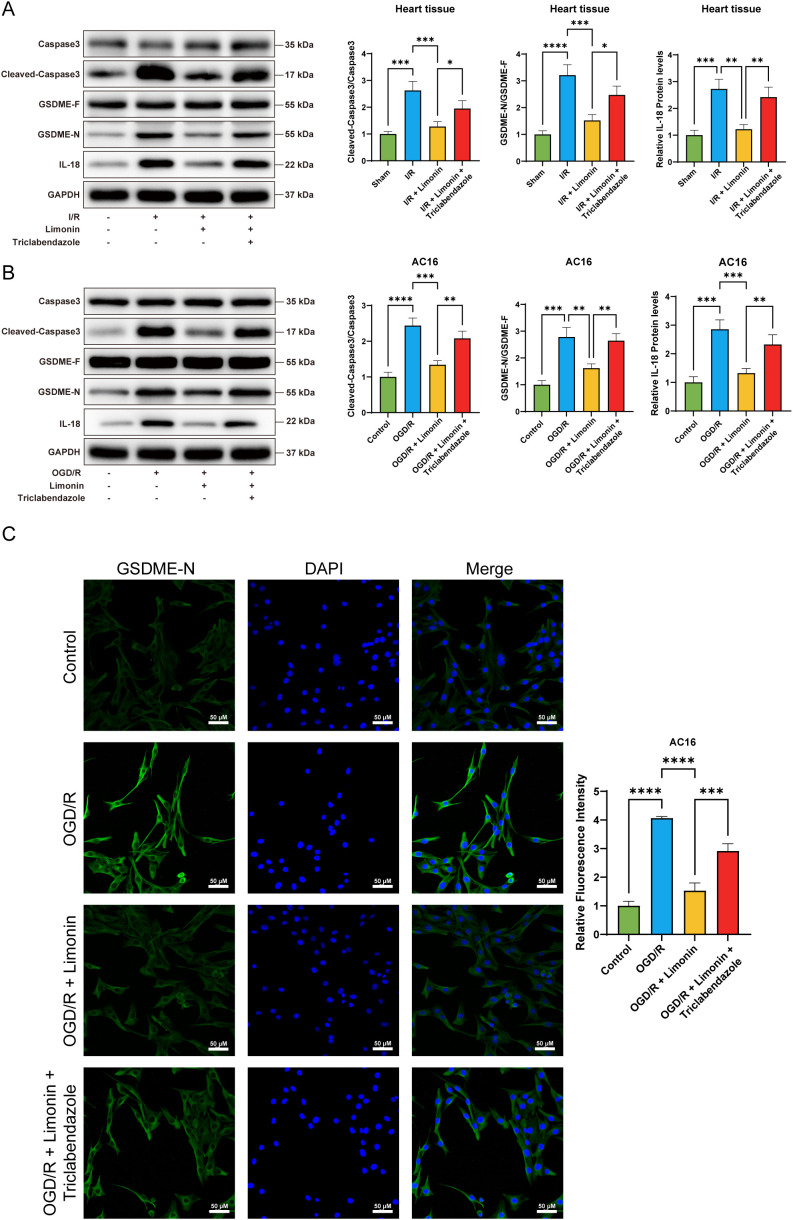
Limonin can inhibit the Caspase-3/GSDME signaling pathway. **(A)** Relative expression levels of caspase-3, cleaved-caspase-3, GSDME-F, GSDME-N, and IL-18 proteins in myocardial tissues detected by western blot experiment (n = 5). **(B)** Relative expression levels of caspase-3, cleaved-caspase-3, GSDME-F, GSDME-N, and IL-18 proteins in AC16 cells detected by western blot experiment (n = 5). **(C)** Detection of GSDME-N expression levels in AC16 cells by immunofluorescence assay (200×, scale bar: 50 μm, n = 5) (*P < 0.05, **P < 0.01, ***P < 0.001, ****P < 0.0001).

The relative expression levels of caspase-3, cleaved-caspase-3, GSDME-F, GSDME-N, and IL-18 proteins in mouse myocardial tissues and AC16 cells were detected using western blotting to explore the effects of limonin and triclabendazole on the caspase-3/GSDME pathway. The results showed that the expression levels of the aforementioned proteins were relatively low in the control group, indicating that the caspase-3/GSDME pathway remained quiescent, accompanied by low levels of pyroptosis and inflammatory response. Compared with the control group, the Cleaved-Caspase-3/Caspase-3 ratio was significantly increased in the I/R model group. Meanwhile, the cleavage of GSDME-F to GSDME-N, which possesses pore-forming activity, was enhanced, and IL-18 protein expression was markedly upregulated. These findings suggest that I/R injury activates the caspase-3/GSDME pathway, induces pyroptosis in cardiomyocytes, and exacerbates the inflammatory response (**Figures 5A, B**). Following limonin treatment (I/R model group + limonin), the expression levels of caspase-3 and cleaved-caspase-3 significantly decreased, the cleavage of GSDME-F to GSDME-N was markedly inhibited, and IL-18 protein expression was reduced. This indicates that limonin effectively inhibits the activation of the caspase-3/GSDME pathway, thereby blocking the occurrence of pyroptosis and alleviating the inflammatory response, thus exerting a cardioprotective effect. However, following the combined administration of triclabendazole (I/R model group + limonin + triclabendazole), the expression levels of caspase-3 and cleaved-caspase-3 increased again, the cleavage of GSDME-F to GSDME-N was enhanced, and IL-18 expression was upregulated. This change demonstrates that triclabendazole reverses the inhibitory effect of limonin on the caspase-3/GSDME pathway, leading to the reactivation of this pathway, thereby restoring pyroptosis and the inflammatory response (**Figures 5A, B**). Immunofluorescence staining was performed to further assess the expression level of GSDME-N. The results showed that, compared with the I/R model group, the fluorescence intensity of GSDME-N was significantly reduced in the limonin treatment group. In contrast, when triclabendazole was included, the fluorescence intensity of GSDME-N was markedly elevated, consistent with the enhanced cleavage of GSDME observed by western blotting (**Figure 5C**). These immunofluorescence findings corroborate the western blot results, further confirming that limonin inhibits the caspase-3/GSDME pathway, whereas triclabendazole reverses this inhibitory effect, thereby regulating pyroptosis and the inflammatory response.

**Figure 6:**
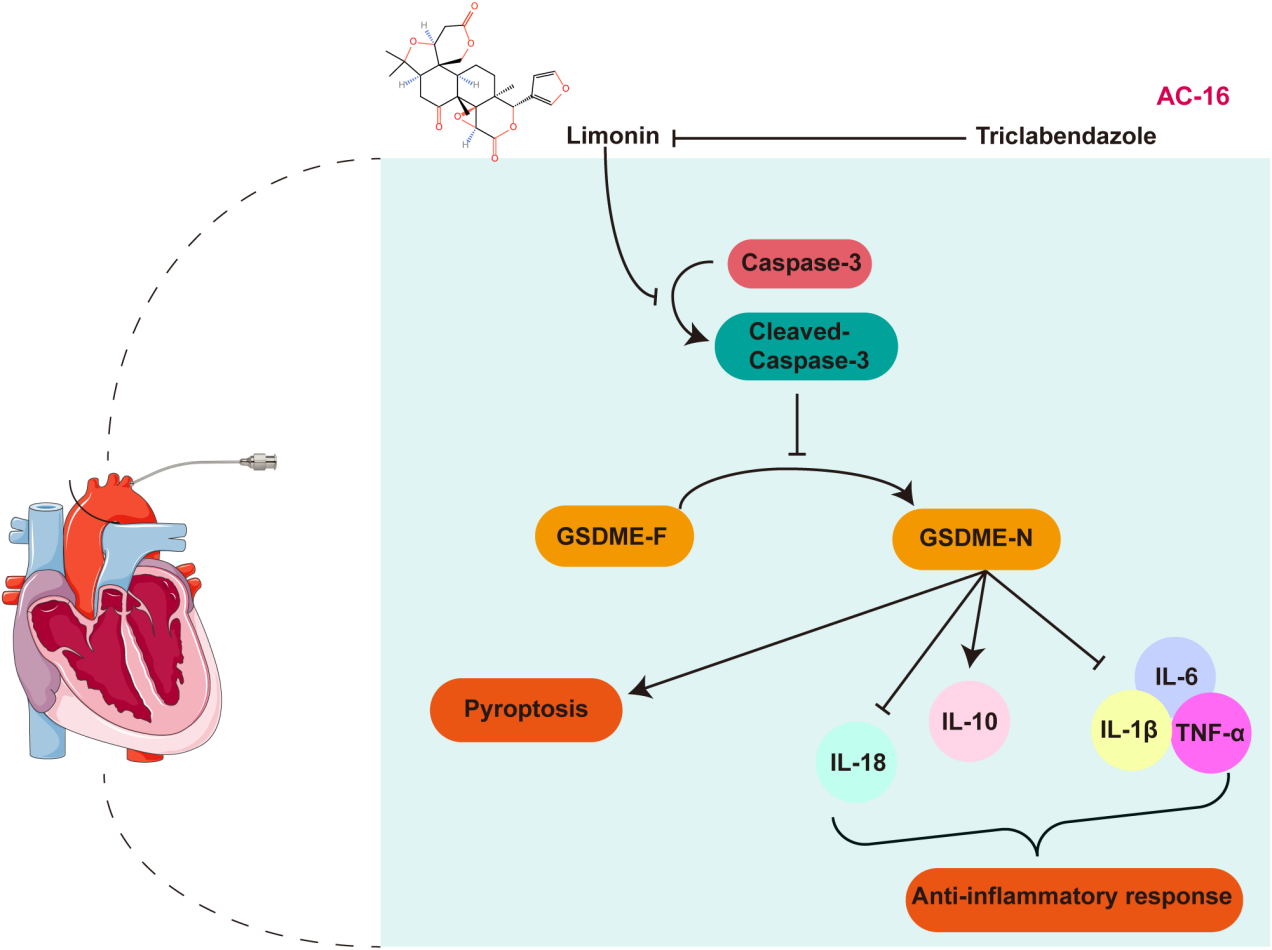
Mechanism diagram of the action pathway of limonin.

Thus, limonin exerted its protective effect by inhibiting the Caspase-3/GSDME pathway, whereas triclabendazole reversed this effect. The specific signaling pathway mechanism is shown in detail in **Figure 6**. The left side of the figure shows the establishment of the I/R model. The right side of the image shows the changes and interactions of a series of regulatory factors in I/R injury. Under normal circumstances, I/R injury leads to the activation of caspase-3 to form cleaved-caspase-3, which then cleaves GSDME-F to form GSDME-N, thereby promoting pyroptosis. At the same time, inflammatory factors such as IL-18, IL-6, TNF-α, and IL-1β are released while the levels of anti-inflammatory factor IL-10 decrease, intensifying the inflammatory response. However, limonin inhibited the activation of caspase-3 and reduced the generation of cleaved-caspase-3, thereby preventing the transformation of GSDME-F to GSDME-N and alleviating pyroptosis. Simultaneously, limonin regulated the balance of inflammatory factors by reducing IL-18, IL-6, TNF-α, and IL-1ß levels and increasing IL-10 production, thereby reducing the inflammatory response. However, the addition of triclabendazole reversed the effects of limonin, leading to the recurrence of pyroptosis and inflammatory responses.

## 4 Discussion

During a review of the literature related to I/R, we noted that the JAK/STAT pathway is considerably complex and plays a dual role in this disease. Some studies indicate that activation of this pathway can have a therapeutic effect (30, 31), while others suggest that inhibition of this pathway has a therapeutic effect (32, 33). Given the complexity of the situation, we shifted the focus of our research to caspase-3 for subsequent experiments. Caspase-3 plays a key regulatory role in the pyroptosis signaling pathway. It is noteworthy that the classical pyroptosis pathway is typically mediated by caspase-1/4/5/11 cleaving GSDMD, primarily in response to inflammasome signaling activated by damage-associated molecular patterns (DAMPs). In contrast, GSDME is specifically cleaved by the apoptosis executioner caspase-3, thereby converting apoptotic signals into pyroptotic signals (34). This mechanistic difference suggests that under pathological conditions characterized by apoptosis and oxidative stress, the mode of cell death may shift from the traditional inflammasome-GSDMD axis to the caspase-3-GSDME axis. This switch depends on the expression level of GSDME and the degree of caspase-3 activation. Although both pathways ultimately lead to cell membrane perforation and the release of inflammatory factors, the upstream activating signals are fundamentally distinct: GSDMD pyroptosis is primarily triggered by extracellular danger signals, whereas GSDME pyroptosis originates from the activation of the intracellular apoptotic program (35–37). Our study found that cleaved fragments of GSDME were significantly increased in the I/R model group, indicating that the switch from apoptotic to pyroptotic signals indeed occurs in MIRI, with caspase-3 activation playing a central driving role. This hypothesis requires further experimental validation. Notably, there is a complex interplay between the two pathways: caspase-3 cannot only cleave GSDME to induce pyroptosis but also cleave the activated GSDMD-NT fragment to inactivate it, thereby inhibiting GSDMD-mediated pyroptosis. This reveals a precise balance mechanism between the apoptosis and pyroptosis pathways (38, 39). In this study, treatment with limonin effectively reversed the I/R-induced activation of caspase-3 and the increase in GSDME cleavage, suggesting that limonin may exert its effects by regulating the activity level of caspase-3. Furthermore, the dual role of caspase-3 as a key node in the cell death network warrants attention: traditionally viewed as a marker of apoptosis, this study indicates that in the presence of GSDME, caspase-3 activation can also trigger pyroptosis. This “apoptosis-pyroptosis switch” phenomenon has not received sufficient attention in the field of MIRI. Future research could clarify the dynamic changes of GSDMD and GSDME at different stages of MIRI as well as the cleavage of GSDMD-NT by caspase-3.

Building on these mechanistic insights, we further investigated the impact of limonin on the inflammatory response during myocardial I/R. Limonin inhibits the inflammatory response induced by myocardial I/R Through a series of experiments, we found that limonin can reduce the levels of pro-inflammatory factors (such as IL-6, IL-1β, and TNF-α) and increase those of the anti-inflammatory factor IL-10, thereby reducing the inflammatory response. In the current global research climate, many studies have focused on the inflammatory mechanisms and potential therapeutic strategies in I/R injury (40, 41). Many natural compounds have been found to have anti-inflammatory properties and diverse mechanisms of action, including the regulation of oxidative stress and inhibition of inflammatory signaling pathways (42–44). Compared to these studies, this study further revealed the unique role of limonin in the inflammatory response to I/R. Our results show that limonin exerts its anti-inflammatory effects by inhibiting the caspase-3/GSDME pathway. This finding is in line with previous studies on the mechanism by which natural compounds reduce inflammation by regulating apoptosis-related pathways (42, 45, 46). However, the effects of limonin on this specific pathway have not been fully elucidated, thus providing a new target for the treatment of I/R inflammation. In addition, our study found that triclabendazole reversed the pharmacological effect of limonin, suggesting a complex network and interaction of multiple factors in the process of inflammation regulation. Future studies should further explore the specific interaction mechanism between limonin and the caspase-3/GSDME pathway and clarify its key sites of action and regulatory links. In addition, the combined application of limonin and other known anti-inflammatory drugs or treatment methods should be studied to provide a more optimized treatment plan for the clinical treatment of I/R-related inflammatory responses.

Overall, this study reveals the important role and potential mechanism of action of limonin in reducing myocardial I/R injury. However, several limitations should be acknowledged. First, although we conducted multifaceted studies at both cellular and animal levels, the translational relevance of our findings is constrained by inherent species differences between rodent models and humans; thus, the role of limonin in the human body requires further clinical trial verification. Second, this study primarily focused on the caspase-3/GSDME pathway; the effects exerted by limonin through other pathways as well as its potential impact on the crosstalk between different cell death modalities, warrant further exploration. Moreover, we utilized only triclabendazole as the pathway activator to validate our mechanistic hypothesis; while informative, the use of a single activator may not fully capture the complex regulatory networks and compensatory mechanisms operating *in vivo*. It should also be noted that TUNEL staining labels DNA fragments and is not exclusively specific to apoptosis, as DNA damage may also occur during the late stages of pyroptosis; therefore, this method has inherent methodological limitations. Future studies should expand our comprehensive understanding of the mechanism of action of limonin and provide a solid theoretical basis for its clinical application.

## 5 Conclusion

Our study provides considerable evidence that limonin exerts a protective effect against myocardial I/R injury by inhibiting the caspase-3/GSDME pathway. This inhibition reduces pyroptosis and inflammation, thereby alleviating cardiac tissue damage and improving cardiac function. Our results indicated that limonin is a potential therapeutic agent for the treatment of I/R-related cardiac injuries. Nevertheless, further studies are required to explore other possible pathways of limonin activity and to verify its efficacy in clinical settings. Furthermore, the complex interactions between limonin and other signaling pathways need to be investigated to fully understand its therapeutic potential.

## Data Availability

The original contributions presented in the study are included in the article/**Supplementary Material**. Further inquiries can be directed to the corresponding author.
